# Rethinking Lafontaine Criteria: Second Metacarpal Cortical Percentage as a Reliable Predictor of Distal Radius Fracture Instability

**DOI:** 10.1177/15589447251346859

**Published:** 2025-06-12

**Authors:** Clinton J. Ulmer, Luke Verlinsky, Chimobi C. Emukah, Mallory J. Ogburn, Bryan Ubanwa, Brian W. Sager

**Affiliations:** 1 The University of Texas Health Science Center at San Antonio, USA

**Keywords:** osteoporosis, wrist instability, wrist, fracture/dislocation, diagnosis, distal radius, fracture/dislocation, diagnosis, fracture closed reduction

## Abstract

**Background:**

Distal radius fractures represent a significant cause of morbidity and loss of independence, particularly in older patients. There is no good consensus on which fractures managed nonoperatively will be unstable in a cast or splint other than Lafontaine criteria, the only relevant clinical study to date. Second metacarpal cortical percentage (2MCP) has been shown to be a reliable predictor of osteoporosis and poor bone quality. This study investigates the utility of 2MCP as an independent predictor of fracture displacement in nonoperative fracture management.

**Methods:**

A retrospective cohort of distal radius fractures treated conservatively over 7 years (2013-2020) was investigated. Injury, postreduction, and 4-week follow-up radiographs were reviewed for 2MCP, volar tilt, ulnar variance, and other demographic factors. Multivariate regression analysis was used to predict fracture displacement.

**Results:**

Only 2MCP and initial fracture displacement were associated with displacement at 4 weeks (*P* = .008, *P* = .008). Other than initial fracture displacement, Lafontaine criteria were not associated with radiographic outcomes. A 2MCP threshold of 53.5% optimized sensitivity (67.5%) and specificity (70.2%) in predicting 10° of fracture displacement (*P* = .003). A 2MCP threshold of 49.5% was 86.7% sensitive and 74.7% specific at detecting dorsal malunion (*P* = .032).

**Conclusions:**

Second metacarpal cortical percentage is a useful clinical tool in predicting distal radius fracture instability. Clinicians can use 2MCP both in guiding decision-making when selecting patients who may benefit from operative management and as a screening tool for osteoporosis and initiation of antiresorptive therapy.

## Introduction

Distal radius fractures (DRFs) are the most common fractures and account for up to 18% of fractures in the geriatric population.^1^ Distal radius fractures represent a significant cause of morbidity and loss of independence, particularly in older patients.^1-3^ These injuries are most commonly managed conservatively with closed reduction and cast immobilization or surgically with open reduction and internal fixation. Previous literature suggests a correlation between decreased bone mineral density (BMD) and the incidence and severity of DRFs.^2,4^ In addition, osteopenia is an independent risk factor for failure of conservative management of these fractures.^5,6^ In 1989, Lafontaine et al^7^ proposed a set of 5 criteria to predict loss of reduction with nonoperative management. Based on the Lafontaine criteria, fractures with 3 or more instability risk factors (age above 60, dorsal comminution, initial displacement >20°, intra-articular extension, ulnar fracture) were more likely to displace with conservative management. Recent systematic review has demonstrated that most literature regarding DRFs has struggled to define “instability” regarding fracture pattern.^8^ To date, only Lafontaine initial article was based on clinical data.

The benefit of operative management for DRFs sustained by the elderly has been a topic of much controversy. A recent meta-analysis suggests that there are no clinically significant functional outcome differences between operative and nonoperative management in patients above 60 years.^9^ Other literature explores the variability between chronologic age and physiological age of individuals, suggesting that older adults with higher functional demands may benefit from operative management.^10^ In addition, a prospective study in patients up to 75 years of age has demonstrated that patients with poor radiographic outcomes, particularly abnormal volar tilt, have diminished function.^11^

Second metacarpal cortical percentage (2MCP) is the proportion of second metacarpal cortical width to total metacarpal width at the midpoint of the metacarpal on posteroanterior (PA) hand radiographs. This measure has been proven to be a useful and precise screening tool for osteoporosis.^12^ A cutoff 2MCP of 50% was 100% sensitive and 91% specific for differentiating osteoporotic patients from normal controls assessed with dual-energy x-ray absorptiometry, the current gold standard in osteoporotic screening.^12,13^ Ghodasra et al^14^ demonstrated that patients with 2MCP less than 50% who underwent conservative management for DRFs were at risk of healing with 1 mm of increased ulnar variance compared with those with normal 2MCP.

Given the inconsistency in the literature in describing fracture characteristics associated with instability and the growing evidence that radiographic outcomes may influence patient function, this study looks to investigate the value of 2MCP and other instability risk factors in predicting radiographic outcomes. We seek to answer 2 questions regarding the nonoperative management of DRFs: (1) Is 2MCP an independent predictor of fracture displacement; and (2) is Lafontaine criteria a valid paradigm for predicting instability and can it be improved?

## Methods

### Patient Selection

This retrospective cohort study reviewed all patients who sustained DRFs identified through a level 1 trauma center’s trauma registry over 7 years (2013-2020). Volar Barton fractures and fractures treated operatively within 2 weeks of injury were excluded. Patients without 4 weeks of radiographic follow-up were excluded. Patient demographics such as age, sex, race, body mass index, and insurance status were recorded. Injury characteristics such as mechanism, open injury, Arbeitsgemeinschaft für Osteosynthesefragen/Orthopaedic Trauma Association (AO/OTA) classification, and Injury Severity Score (ISS) were also recorded.

### Radiographic Measurement and Variable Definition

**Figure 1.**
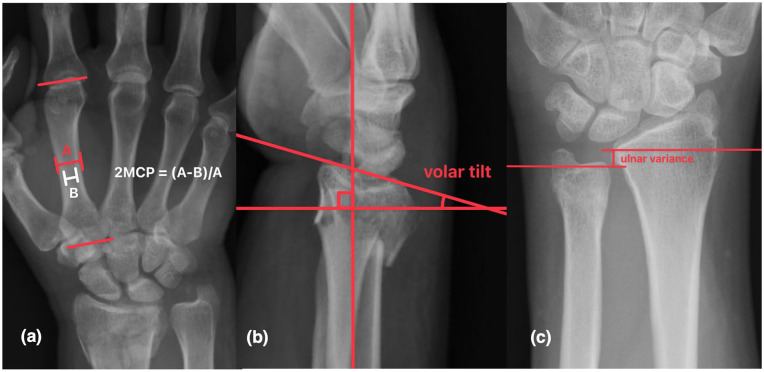
Measurement of (a) 2MCP, (b) volar tilt, (c) ulnar variance. 2MCP = second metacarpal cortical percentage.

Injury, postreduction, and 4-week follow-up wrist and hand radiographs were reviewed by 2 senior orthopedic surgery residents. Lafontaine criteria were also denoted, including age, dorsal comminution, intra-articular involvement, initial dorsal displacement, and associated ulnar styloid fracture. Second metacarpal cortical percentage, volar tilt, and ulnar variance were measured. Second metacarpal cortical percentage was defined as the proportion of second metacarpal total cortical width to total metacarpal width at the midpoint of the metacarpal on PA hand radiographs (Figure 1a). Volar tilt was measured as the angle between a line connecting the volar and dorsal rim of the lunate facet, and a line perpendicular to the axis of the radius on lateral radiographs (Figure 1b). Ulnar variance was assessed as the height difference between the ulnar fovea and the ulnar volar rim of the lunate facet on PA wrist radiographs (Figure 1c). Fracture displacement in the sagittal plane was calculated as the difference between volar tilt at 4 weeks and immediate postreduction. Given no contralateral films were readily available, initial fracture displacement in the sagittal plane was estimated as the difference between the volar tilt on initial radiograph and the average anatomic volar tilt (11°). Fracture shortening was defined as the difference between ulnar variance at 4 weeks and immediate postreduction radiographs. Malunion was defined as a dorsal tilt >20° at 4 weeks. Fracture displacement in the sagittal plane was selected as the primary outcome of interest, with secondary outcomes of fracture shortening and malunion.

### Statistical Models

Univariate linear regression models were used to initially screen for continuous effect modifiers and confounders. Independent-sample *t* tests and Wilcoxon rank-sum tests were performed for normally and non-normally distributed data, respectively. Initially, 2 multivariate linear regression models predicting Δvolar tilt were developed, one using covariates in the original Lafontaine criteria (age, dorsal comminution, initial displacement, intra-articular extension, ulnar styloid fracture) and a new model replacing age with 2MCP. Akaike information criterion (AIC) statistics were computed for comparison of model fit. A third simplified model with only statistically significant covariates (2MCP, initial displacement) was tested, identified as the best fit by AIC, and used for the remainder of analyses. Residual plots were analyzed to ensure appropriateness of the linear regression model. This was repeated for the secondary outcome of Δulnar variance.

Subsequently, a multivariate logistic regression model predicting 10° of fracture displacement with covariates of 2MCP and initial displacement was selected and compared with a model with the original Lafontaine criteria. Receiver operating characteristic (ROC) curves were computed, and area under the curve (AUC) analysis was performed. The optimal 2MCP cutoff for predicting sagittal plane fracture displacement was calculated using the Youden *J* test maximizing sensitivity and specificity. This was repeated for the secondary outcome of malunion. Significance level was set as .05 a priori. Statistical analysis was performed using R 4.3.1 (Posit PBC, Boston, Massachusetts) with the *pROC* (v1.18.4) and *ggplot2* (v3.4.3) packages.^15-17^

## Results

A total of 377 DRFs treated nonoperatively with closed reduction and casting were identified within the trauma registry. Of 377 patients, 126 fractures in 123 patients with a minimum of 4 weeks of radiographic follow-up were identified. Patient age ranged from 19 to 84 years with a mean of 54.2 years. About 54% of patients were men. Most fractures (58.6%) were sustained in high-energy trauma mechanisms (motor vehicle collisions, falls from height, and motor pedestrian collisions), with 63.5% of patients sustaining AO/OTA type C fractures. Demographic data are presented in Table 1. Second metacarpal cortical percentage values ranged from 28.4% to 88.4%.

**Table 1.** Descriptive Statistics.

Variables	N = 126
	No. (%)/mean (SD)
Age, y, mean (SD)	54.2 (17.6)
Male	68 (54.0%)
BMI, kg/m^2^, mean (SD)	28.8 (6.88)
Insurance status	
Private	34 (27.0%)
Medicare	32 (25.4%)
Worker’s compensation	7 (5.6%)
Medicaid	6 (4.8%)
Uninsured	47 (37.3%)
Mechanism of injury	
Mechanical ground-level fall	50 (39.7%)
Motor vehicle collision	28 (22.2%)
Fall from >3 m	24 (19.0%)
Vehicle pedestrian collision	10 (7.9%)
Motorcycle collision	9 (7.1%)
Blunt trauma	2 (1.6%)
Bicycle	2 (1.6%)
Crush	1 (0.8%)
Open fracture	2 (1.6%)
AO/OTA classification	
23A	35 (27.8%)
23B	11 (8.7%)
23C	80 (63.5%)

*Note*. All values presented as count (percentage) unless otherwise specified. AO/OTA = Arbeitsgemeinschaft für Osteosynthesefragen/Orthopaedic Trauma Association. BMI = body mass index.

**Figure 2.**
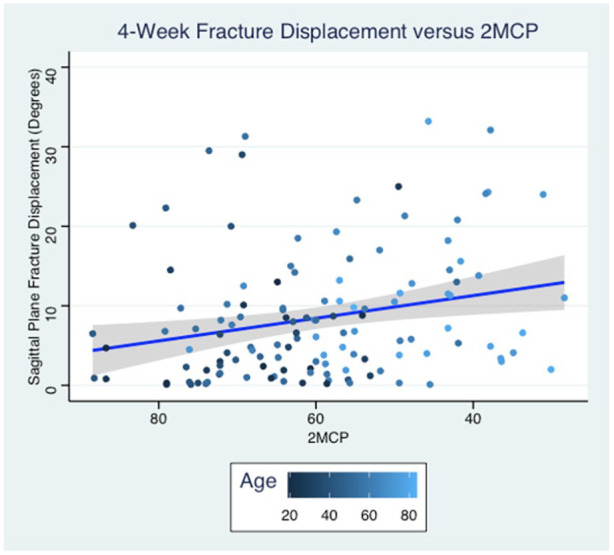
Linear regression model comparing 2MCP to fracture displacement at 4-week follow-up. 2MCP = second metacarpal cortical percentage.

In a linear regression model using Lafontaine original 5 risk factors, only initial displacement was associated with sagittal plane fracture displacement (*P* = .019). Age, dorsal comminution, intra-articular extension, and ulnar fractures were not associated with radiographic outcomes at 4 weeks (Table 2). In a model replacing age with 2MCP, 2MCP and initial fracture displacement were found to have statistically significant associations with fracture displacement (*P* = .008, *P* = .008; Figure 2). No tested variables had statistically significant predictive value regarding fracture shortening.

**Table 2.** Linear Models Predicting 4-Week Fracture Displacement in Sagittal Plane.

Model	*P* value	AIC	*R* ^2^
Lafontaine criteria		878	0.064
Age	.084		
Intra-articular extension	.175		
Dorsal comminution	.667		
Ulnar fracture	.957		
Initial displacement	.019		
Modified Lafontaine criteria		874	0.096
2MCP	.008		
Intra-articular extension	.172		
Dorsal comminution	.714		
Ulnar fracture	.832		
Initial displacement	.008		
Final model		870	0.102
2MCP	.005		
Initial displacement	.006		

*Note*. AIC = Akaike information criterion; 2MCP = second metacarpal cortical percentage.

**Figure 3.**
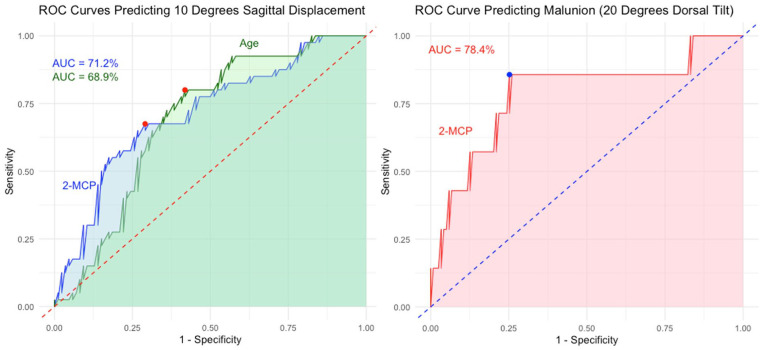
ROC curves (a) comparing 2MCP and age in predictive model of 4-week fracture displacement of 10°. (b) 2-MCP in predictive model of final fracture alignment of 20° of dorsal tilt. *Note*. ROC = receiver operating characteristic; 2MCP = second metacarpal cortical percentage; AUC = area under the curve.

A 2MCP threshold of 53.5% optimized sensitivity (67.5%) and specificity (70.2%) in predicting 10° of fracture displacement, with area under the ROC curve of 71.2% (*P* = .003). A 2MCP threshold of 49.5% optimized sensitivity (86.7%) and specificity (74.7%) in predicting dorsal malunion, with area under the ROC curve of 78.4% (*P* = .032; Figure 3; Table 3).

**Table 3.** ROC Curve Analysis of 2MCP Cutoff Values.

Outcome	2MCP cutoff
10° fracture displacement	53.5
Final dorsal tilt >20°	49.5

*Note*. ROC = receiver operating characteristic; 2MCP = second metacarpal cortical percentage.

## Discussion

Fractures involving the distal radius account for one sixth of all fractures and can cause significant morbidity and loss of independence particularly in the elderly population.^1,2^ Our findings support the current evidence that decreased BMD is associated with nonoperative treatment failure.^5,6^ Patients with less than 50% 2MCP have a high likelihood of being osteoporotic.^12^ Using a different set of radiographic criteria on injury films, Lafontaine showed that certain risk factors increased the likelihood of loss of reduction, criteria that are often used to guide management. Our study demonstrates that use of 2MCP and initial fracture displacement alone was more reliable than Lafontaine criteria for fracture instability in the sagittal plane.

Measurement of 2MCP on injury radiographs in patients with a DRF, therefore, can be helpful in counseling patients on operative and nonoperative management options for their fractures. Recent studies on patients above 65 years of age have found that functional outcomes are initially improved with operative intervention, although similar at 1 year after injury.^18^ Schmidt et al demonstrated in a prospective study that dorsal tilt over 20° after DRF is associated with loss of grip strength and decreased range of motion. The risk of malunion and the potential for diminished functional outcomes combined with 2MCP measurements should be brought up with patients and families during shared decision-making discussions. With the increasing prevalence of osteoporosis and a growing geriatric population, it is crucial to identify patients who may benefit from early operative intervention to prevent dorsal malunion. Our study data suggest that a 2MCP cutoff of approximately 50% is 87% sensitive and 75% specific in detecting patients with a dorsal malunion of 20° or more.

In addition to predicting instability, this study redemonstrates the validity of 2MCP as a clinical tool in screening for osteoporosis. Almost all patients with DRFs present with hand radiographs. Any fragility fracture, including DRFs, is considered a major risk factor for future fragility fractures. Using 2MCP after a DRF, orthopedic surgeons are in a unique position to initiate early anti-osteoporotic therapy in older patients with osteoporosis.^19-21^ Initiating treatment with anti-osteoporotic therapy after a fragility fracture leads to a 40% decrease in the 3-year risk of subsequent fracture.^19^ Implementation of fragility clinics or referral to fracture liaison services has been shown to be effective in fracture prevention.^3^ Measurement of 2MCP can be used both to counsel patients on best treatment for their injuries and to identify those who would benefit from early intervention to reduce the risk of future fragility fractures.

There are several limitations of our study. Foremost, the predictive model presented has a high amount of variance unaccounted for by the risk factors studied. The sample of patients in our study was retrieved from a trauma registry and was skewed toward more severe injuries and high-energy mechanisms than the general population. Despite this, we find that our model adds value to the current evidence, as there is great heterogeneity in the current definition of instability in DRF literature. We propose that 2MCP is a more useful metric than age as originally reported by Lafontaine. Conceptualizing biologic age and frailty in predicting orthopedic outcomes as opposed to chronologic age alone is paramount in clinical decision-making.^10,22^ Further studies on functional outcomes in geriatric DRFs in relation to radiographic outcomes are warranted, including evaluations of more generalized populations, including patients not included in a trauma registry. In addition, further investigations into the interobserver reliability of 2MCP in multiple age groups should be performed to further validate its use in providing a quantitative value of osteopenia throughout a broader group of patients.

Second metacarpal cortical percentage is a valuable clinical tool in both screening for osteoporosis and predicting DRF instability, with a 50% cutoff value for stratifying risk of fracture displacement in the sagittal plane. Using 2MCP as an additional piece of data when considering operative or conservative management of patients may be valuable, as dorsal collapse and malunion can lead to functional deficits, even in the geriatric population. Hand radiographs can be used as a useful screening tool for referral to fragility clinics or the start of antiresorptive therapy to prevent further fragility fractures.
